# Neural Stem Cells: What Happens When They Go Viral?

**DOI:** 10.3390/v13081468

**Published:** 2021-07-27

**Authors:** Yashika S. Kamte, Manisha N. Chandwani, Alexa C. Michaels, Lauren A. O’Donnell

**Affiliations:** Graduate School of Pharmaceutical Sciences, School of Pharmacy, Duquesne University, 600 Forbes Avenue, Pittsburgh, PA 15282, USA; kamtey@duq.edu (Y.S.K.); chandwanim@duq.edu (M.N.C.); michaelsa@duq.edu (A.C.M.)

**Keywords:** neural stem cells, viruses, proliferation, survival, neurogenesis, differentiation, gliogenesis, cell death, cytokines

## Abstract

Viruses that infect the central nervous system (CNS) are associated with developmental abnormalities as well as neuropsychiatric and degenerative conditions. Many of these viruses such as Zika virus (ZIKV), cytomegalovirus (CMV), and herpes simplex virus (HSV) demonstrate tropism for neural stem cells (NSCs). NSCs are the multipotent progenitor cells of the brain that have the ability to form neurons, astrocytes, and oligodendrocytes. Viral infections often alter the function of NSCs, with profound impacts on the growth and repair of the brain. There are a wide spectrum of effects on NSCs, which differ by the type of virus, the model system, the cell types studied, and the age of the host. Thus, it is a challenge to predict and define the consequences of interactions between viruses and NSCs. The purpose of this review is to dissect the mechanisms by which viruses can affect survival, proliferation, and differentiation of NSCs. This review also sheds light on the contribution of key antiviral cytokines in the impairment of NSC activity during a viral infection, revealing a complex interplay between NSCs, viruses, and the immune system.

## 1. Introduction

Viral infections in the central nervous system (CNS) are capable of inducing long-term neurological damage, particularly when the immune response fails to fully resolve the infection. Neurotropic viruses can alter the activity of infected neural cells and even lead to cell death. Neural stem cells (NSCs) are the pluripotent stem cells of the brain, and they are vulnerable to infection by numerous neurotropic viruses [1,2,3,4,5,6,7]. NSCs have the ability to both “self-renew” through symmetric cell division and to differentiate into neurons and glial cells. Both of these functions are critical to building the developing brain in pre- and post-natal periods and to maintain physiological function and responses to injury within select regions of the adult brain [8]. NSC proliferation and differentiation is governed by a finely orchestrated series of signals, which when disrupted can result in dysfunction in the development of neural circuits [9]. Thus, diseases that disturb physiological NSC activity can have lifelong consequences, particularly when they occur during key stages of neurodevelopment [10].

Viruses interrupt stem cell function in different ways, depending upon the virus and age of the host. The vulnerability of NSCs to viral infections is often age-dependent, with the younger population displaying more profound effects [10]. The older population does not remain immune to changes in NSC activity, since NSCs in the adult neurogenic niches (e.g., hippocampus and subventricular zone (SVZ)) undergo neurogenesis to govern memory and olfaction [8,11,12,13,14]. However, we are only beginning to understand the viral and host factors that modulate NSC activity. NSCs may be directly infected by viruses, leading to changes in commitment to other cell types and potentially cell death. NSCs can also be affected by cytokines and chemokines that are released as part of the antiviral immune response [15,16,17]. It has been complicated to dissect the contribution of the viral infection and the antiviral immune response in mediating NSC activity, but recent in vitro and in vivo studies have shed light onto how NSCs respond to viruses. In this review, we will focus on the consequences of viral infections of NSCs and the associated outcomes on survival, proliferation, and differentiation.

## 2. NSC Survival and Proliferation

**Figure 1:**
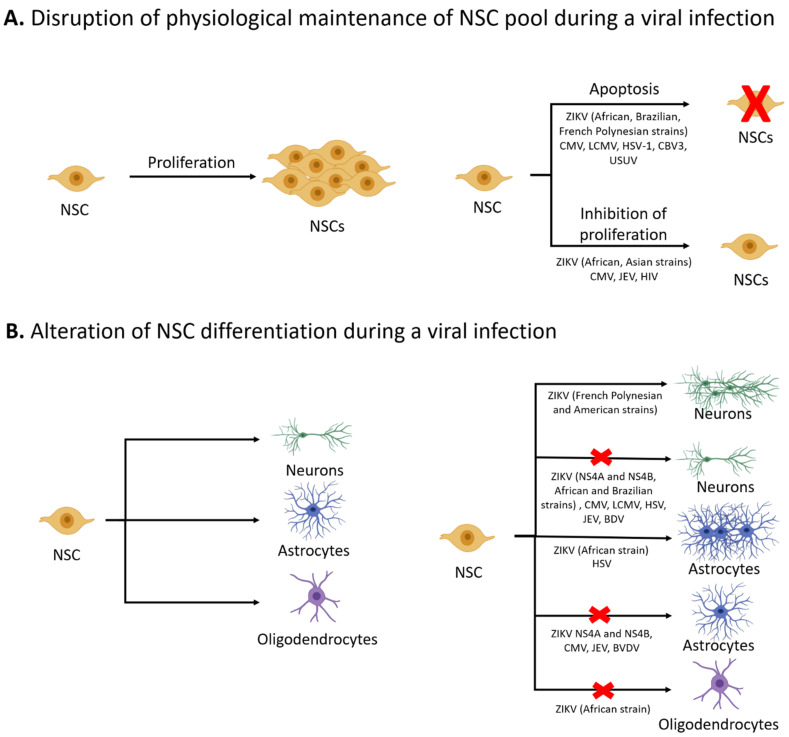
Effects of viral infections on the physiological functions of NSCs. Under physiological conditions, NSCs can proliferate via symmetric cell division to maintain the NSC pool. Viral infections such as ZIKV and CMV can deplete the NSC pool by inducing apoptosis or reducing proliferation of NSCs (**A**) Based upon tightly coordinated developmental cues, NSCs undergo differentiation to yield neurons, astrocytes, or oligodendrocytes. Viral infections can result in aberrant differentiation of NSCs, with many viruses leading to reduced neurogenesis (**B**) Some viruses, such as CMV and JEV, can inhibit both neurogenesis and gliogenesis. ZIKV impacts differentiation in a strain-dependent manner; the ZIKV Brazilian strain (BR_ZIKV_AB_ES) reduced neurogenesis whereas ZIKV French Polynesian strain (H/PF/2013) and ZIKV American strain (FB-GWUH-2016) increased the number of cells expressing a neuronal marker (Tuj1). Viruses can also affect the formation of glia, with many viruses blocking astrogliogenesis and ZIKV African strain (IB H 30656) blocking the formation of oligodendrocytes.

NSCs undergo periods of extensive cell division in order to maintain the NSC pool, and disruptions in proliferation at critical periods in development can lead to an extended loss of NSCs (Figure 1A). Reduction of the NSC pool can limit long-term neurogenesis, which is associated with the development of neurodegenerative diseases and deficits in adult learning and memory [18,19]. NSCs can be depleted due to multiple, non-exclusive mechanisms, including inhibited proliferation, increased cell death, and/or greater commitment to other cell lineages. Viral infections such as Zika virus (ZIKV), herpes simplex virus-1 (HSV-1), lymphocytic choriomeningitis virus (LCMV), and Japanese encephalitis virus (JEV) are associated with a range of neurological outcomes including microcephaly, blindness/hearing loss, memory deficits, and cognitive decline, respectively [3,20,21,22,23,24,25]. Although these viruses vary in terms of cellular tropism and disease course, they are also associated with disruptions in NSC activity, which has implications for life-long neurogenesis in the brain (Figure 1A) [26,27,28]. The underlying mechanisms driving the loss of NSCs remain largely undefined; however, clues are beginning to emerge from the literature.

Reduced proliferation can lead to a depletion of the NSC pool. Viruses may inhibit proliferation by blocking specific stages of the cell cycle and/or modulation of proteins that control self-renewal (Table 1). Many in vitro models of NSC infection (e.g., ZIKV-MR766, cytomegalovirus (CMV), JEV, and human immunodeficiency virus (HIV)) are associated with DNA damage and cell cycle arrest in the S-phase (DNA synthesis phase) of the cell cycle [1,29,30,31,32,33]. During JEV infection, reduced NSC proliferation may also be accompanied with increased neurogenesis, which could further reduce the NSC pool. Some viruses interfere with the expression of NSC-specific regulatory proteins that are required for maintaining a proliferative state. CMV infection of human NSCs suppresses Hes1 expression, which is an important protein for governing NSC cell fate choice and proliferation [34,35]. NSC treatment with HIV tat protein results in nuclear localization of the E3-ubiquitin ligase tripartite containing motif 32 (TRIM-32) protein, accumulation of which is associated with reduced NSC proliferation and increased neurogenesis. These in vitro findings were confirmed in autopsy samples of HIV-seropositive individuals, where reduced NSC numbers were accompanied by increased TRIM-32 nuclear localization in the remaining NSCs [36]. These studies indicate there are multiple mechanisms via which viral infections might lead to reduced NSC proliferation.

NSC numbers can also be depleted due to apoptosis. Numerous viruses such as CMV, JEV, LCMV, West Nile virus (WNV), Usutu virus (USUV), enterovirus 71 (EV71), and Cocksackie B virus (CVB3) induce apoptosis in NSCs [30,31,32,37,38,39,40,41]. Both intrinsic and extrinsic apoptosis pathways can be activated during viral infection [32,42]. CMV and JEV infection induces apoptosis via activation of the MAP-Kinase (MAPK) pathways and the unfolded protein response (UPR) activation, which are triggered through endoplasmic reticulum (ER) stress [4,32,42]. Viral infections can induce ER stress possibly due to the utilization of ER proteins required for immediate early viral replication and translation [43,44]. Whether a virus induces apoptosis or inhibits proliferation may be influenced, in part, by the choice of model system. HSV-1 infection of monolayer cultures results in cell death of the majority of the NSCs [45]. However, HSV-1 infection of NSCs in cerebral organoids or 3D cultures induced mild cell death despite increased expression of genes associated with apoptosis [46]. These variations in NSC survival highlight the importance of considering multiple NSC models for investigating interactions with viruses, including those that recapitulate elements of the neural microenvironment.

Although many viruses are associated with apoptosis in NSCs, the extent of cell death versus cell cycle blockade can be dependent on the strain of the virus [27,47]. ZIKV infections can be associated with multiple effects on NSCs (Figure 1A), which may be partially attributed to differences in viral replication. For instance, ZIKV MR766 and IBH 30656 (African strains) yield high concentrations of viral proteins during infection of human cells, whereas ZIKV PRVABC59 and H/PF/2013 (Asian strains) result in undetectable protein concentrations soon after ZIKV infection [48]. The African ZIKV strains have been shown to cause higher mortality and severe neurological complications in comparison to the Asian ZIKV strains [49]. The ZIKV Paraiba Brazil strain primarily results in apoptosis, is more virulent, and results in excitotoxicity in a mouse model as compared to the Asian H/PF/2013 strain [50]. Similarly, these strains of ZIKV display a spectrum of effects on NSC activity and survival. Infection of NSCs by more pathogenic strains, such as the Brazilian Paraiba strain, leads to activation of the autophagy pathway, possibly to increase viral replication resulting in cell death [51]. Infection of NSCs with the African strains (MR766 and IB H 30656) are associated with more severe NSC cell death [52]. Interestingly, ZIKV-MR766 infection of NSCs caused apoptosis in a Musashi-1 dependent manner [53]. Musashi-1 is a neural RNA binding protein that is key for self-renewal of NSCs and neurogenesis [54]. The interaction between ZIKV-MR766 and a key NSC regulatory protein provides a mechanistic link between the apoptotic effects of the virus and resulting neuropathology. In contrast, the infection of NSCs by the H/PF/2013 strain of ZIKV leads to a mild cytopathic effect [41], while infection with PRVABC59 of ZIKV strain results in cycle arrest and reduced proliferation [55]. These results suggest that the severity of outcomes during viral infections in the brain could be dependent on the virus strain (Table 1) and how those strains impact NSCs.

In some CNS infections, the virus may not be fully cleared or controlled, but may persist in the CNS [56]. NSCs can maintain persistent infections in the surviving pool of cells, as has been observed for ZIKV and CVB3 [57,58]. HIV-1 also can persist in the CNS, potentially serving as a reservoir for the virus. Autopsy samples of HIV-1 patients with dementia revealed a reduction in NSC numbers compared to patients without dementia. Further in vitro studies suggested that the HIV gp120 protein reduced NSC proliferation via disruption of the extracellular signal-regulated kinase (ERK) pathway [59]. HSV-1 infection of adult NSCs results in reduced proliferation and neurogenesis without cell death and accumulation of amyloid-β protein fragments, a biomarker of Alzheimer’s disease [22,60]. While it is tempting to speculate about the potential role of viruses in neurodegenerative disease, these studies highlight that the consequences of viral infections in NSCs can be long-lasting and suggest that persistent viral infections may contribute to development of other neurological conditions.

## 3. Differentiation of NSCs

During brain development, NSCs differentiate in a temporal sequence with neurogenesis occurring first followed by gliogenesis [8,14,61,62,63,64]. In rodents, neurogenesis begins during early embryonic stages 10–11 (E10–E11) and continues till birth, although dendritic and axonal maturation continues postnatally [61,65]. Gliogenesis begins during late embryonic stages and peaks at birth [61,63,64]. In humans, both neurogenesis and gliogenesis occur majorly during gestation, but development continues even after birth [65]. The switch from neurogenesis to gliogenesis during development is tightly regulated by basic helix-loop-helix (bHLH) transcription factors and signaling pathways such as bone morphogenetic proteins (BMPs), Notch, and the janus kinase/signal transducer and activator of transcription (JAK-STAT) pathway [56,66]. In adults, the NSC pool becomes restricted to two neurogenic niches, the dentate gyrus (DG) of the hippocampus and the SVZ [14]. Specifically, NSCs in the DG give rise to glutamatergic excitatory dentate granule cells and contribute to spatial learning and memory [11,67,68,69,70,71,72,73,74]. While in the SVZ, the primary role of this NSC pool is to give rise to neuroblasts that migrate to the olfactory bulb (OB) via the rostral migratory stream (RMS) to support olfactory neurogenesis [71,75,76].

Aberrant differentiation of NSCs is associated with neurodevelopmental, neurodegenerative, and neuropsychiatric conditions. Increased neuronal density is observed in the prefrontal cortex of children with Autism spectrum disorder (ASD), suggesting increased neurogenesis [75,76,77]. In contrast, reduced neurogenesis, and a consequent decrease in number of mature neurons in both the hippocampus and SVZ, is seen in a mouse model of Parkinson’s Disease (PD) with overexpression of human wild-type alpha synuclein [78,79]. Schizophrenia is also associated with reduced neurogenesis specifically in the hippocampus and prefrontal cortex [80]. These studies highlight that alterations in NSC differentiation may have functional consequences in development of the brain and of neurological disease.

Neurotropic viruses, including ZIKV, CMV, HSV, and borna disease virus (BDV) among others, can also alter NSC differentiation (Table 1) [81,82]. Viral infections can shift NSC fate toward the neuronal or glial lineage, sometimes at the expense of the other lineage or with no apparent impact on the other lineage. Viruses can also dampen differentiation broadly by inhibiting the formation of multiple neural cell types (Figure 1B). For example, HSV infection of NSCs decreases neuronal differentiation and shifts fate to the glial lineage [46,60], while BDV infection decreases neuronal differentiation with no impact on the glial lineage [2,83]. Commitment to the astroglial lineage is blocked by bovine viral diarrhea virus (BVDV) infection, whereas no changes were seen in the neuronal or oligodendroglial lineages [84]. JEV and CMV can inhibit both neuronal and glial differentiation, although Kosugi et al. showed that CMV inhibits neuronal lineage more profoundly [1,5,30,31,85]. As observed with NSC proliferation, the impact of ZIKV on differentiation is strain-dependent. The ZIKV Asian strain (H/PF/2013) and American strain (FB-GWUH-2016) are seen to lead to premature neuronal differentiation, while the ZIKV African (IBH 30656) and Brazilian (BR_ZIKV_AB_ES) isolates were seen to inhibit neuronal differentiation. The ZIKV African isolate (IBH 30656) showed upregulation of a number of astrocytic genes, indicating that fate of NSCs might shift to the astroglial lineage [52,86,87,88]. These studies show that the impact of ZIKV infection on NSC differentiation is not monolithic and varies by strain and perhaps partly by the model system as well. Thus, viruses disrupt differentiation in multiple ways, including inhibition of a single lineage, shifting into alternative lineages, or broadly dampening differentiation into multiple cell types.

The exact mechanisms by which viruses impair NSC differentiation are not well understood. However, one could imagine that both viral proteins and cellular factors would contribute to changes in differentiation. Specific viral proteins from ZIKV and BDV have been identified that impair differentiation. The BDV phosphoprotein P, which is a part of the polymerase complex, is responsible for decreasing neuronal differentiation via downregulation of multiple neuronal genes [83]. Similarly, ZIKV non-structural (NS) proteins NS4A and NS4B, involved in viral replication and immune evasion, lead to reduction of both neuronal and astrocytic numbers [89,90,91]. Specifically, NS4A and 4B, whether expressed individually or together, were shown to inhibit the Akt-mTOR signaling pathway, which is important for neurogenesis and brain development [91,92,93]. The inhibition of the Akt-mTOR signaling also enhanced autophagy, which may support ZIKV replication as well as contribute to loss of the NSC pool [94,95,96]. These examples highlight that certain viral proteins are central to dysregulation of NSC differentiation.

The modulation of cellular proteins by the infection and subsequent immune response also contribute to aberrant differentiation. The JAK/STAT pathway is key to mediating the switch between neurogenesis and astrogliogenesis. During neurogenesis, the bHLH transcription factors inhibit the expression of the JAK-STAT proteins in order to prevent premature formation of astrocytes. Downregulation of the bHLH factors leads to leukemia inhibitory factor (LIF)-mediated induction of the JAK/STAT-1/3 pathway, which triggers astrogliogenesis [66,97]. JEV infection of NSCs reduces JAKI expression and STAT3 phosphorylation and increases STAT1 phosphorylation, thereby impairing astrocytic differentiation [1]. JEV also inhibited neuronal differentiation through downregulation of multiple neuronal genes (Ngn1, Ngn2, and NeuroD1) [1]. CMV similarly impairs neurogenesis, but through a distinct mechanism involving the peroxisome proliferator-activated receptor-γ (PPARγ). PPARγ is a ligand dependent transcription factor that is highly expressed in the embryonic brain. In NSCs, PPARγ plays multiple roles in differentiation, including promotion of astrogliogenesis and inhibition of neurogenesis [85,94]. During CMV infection, PPARγ levels are elevated in the fetal brain. In vitro studies showed that PPARγ expression increased in CMV-infected NSCs and is associated with less neuronal differentiation [85,98]. Treatment of CMV-infected NSCs with a PPARγ inhibitor rescued abnormal differentiation, demonstrating the potential of identifying druggable targets for restoring NSC function.

The stage of NSC maturation is an important variable that determines susceptibility to infection and subsequent impacts on differentiation. Undifferentiated stem cells are often more permissible to viral infections and subsequent cell death, perhaps due to mounting a poor innate immune response as noted by the lower expression of Toll-like receptors, STAT1/2, and interferon-stimulated genes (ISGs) in undifferentiated NSCs versus more differentiated cells [1,99]. ZIKV infection of undifferentiated cells showed a greater cytopathic effect as compared to cells that have initiated astrocytic differentiation [55]. In differentiating NSCs, CMV decreased neuronal numbers only when infection occurred in the first 24 h after induction of differentiation; CMV infection at later time points did not impact neuronal differentiation [30]. These studies suggest that many viruses favor immature or undifferentiated NSCs, leading to greater viral infection but also more severe impacts on differentiation.

In addition to the virus, antiviral cytokines and chemokines can also disrupt NSC differentiation [15]. Chemokines orchestrate the movement of immune cells to the site of injury or infection, and chemokine receptors are often expressed on NSCs [95]. In a mouse model of congenital LCMV infection, persistent infection was associated with the expression of several chemokines (e.g., CCL2, CCL5, CXCL9, and CXCL10) in the neurogenic niches of the brain [37]. Persistent LCMV infection also reduced neuroblasts in the adult brain, which could be associated with the upregulation of these chemokines in the same regions [37]. Similarly, neonatal LCMV infection was associated with a decrease in adult neurogenesis in the DG, indicating that infections in the both the fetal and neonatal CNS could have long-lasting consequences [96]. Cytokines can also impact NSC differentiation. IL-1β impaired neurogenesis after WNV infection in adult mice and has been shown to have pro-gliogenic and anti-neurogneic effects on NSCs in vitro [100,101,102]. IFNγ is a key antiviral cytokine that can modulate NSC activity [103,104]. In chronic HSV-1 infection, CD8+ T cells are the major source of IFNγ in the brain. In vitro co-culture of CD8+ T cells and NSCs shifted differentiation towards the astroglial lineage in an IFNγ-dependent manner [105]. Together, these findings suggest that viruses can disrupt NSC differentiation through expression of viral proteins, modulation of cellular factors, and the induction of cytokines and chemokines by the antiviral immune response.

## 4. Conclusions

NSCs are critical for the extended development of the brain and for plasticity and repair in the mature CNS [106]. It is apparent that many neurotropic viruses are capable of disturbing NSC function, with profound consequences for the host in some instances. However, a number of outstanding questions remain regarding interactions between viruses and NSCs. Although changes in NSCs have been observed at the cellular level, how such changes contribute to neurological and psychiatric disorders is a complex question. Damage from the acute infection and subsequent long-term alterations in the cellular organization of the brain are possible outcomes. Connections between neurotropic infections and neurodegenerative disease later in life, such as the relationship between HSV-1 infections and Alzheimer’s disease, are currently being explored. Nevertheless, it is clear that the age of the host and the maturation of the NSCs are key variables in how viruses impact NSCs. TORCH pathogens [toxoplasmosis, other pathogens (e.g., syphilis, parvovirus), rubella, CMV, HSV], which are associated with congenital infections of the fetus or newborn, can cause catastrophic dysregulation and depletion of NSCs, contributing to a long-term loss of both neural progenitors and new neurons associated with microcephaly (see [107] for a comprehensive review). In a model of adult WNV infection, cytokine-mediated inhibition of hippocampal neurogenesis is associated with memory impairments, even though the number of NSCs remained stable [100].

As mechanisms of NSC dysfunction are being explored, it is important to consider the contributions of both the virus and the antiviral immune response. Due to overlap in signaling pathways between developmental and inflammatory cytokines (e.g., STAT signaling), cytokines produced in response to the virus can shift commitment of NSCs between neuronal and glial lineages. In vitro studies provide powerful tools for capturing interactions between NSCs and viruses or specific cytokines, which enhance our understanding of the factors that may influence cellular changes in vivo. However, it is ultimately necessary to assess the combination of the viral infection and signaling factors produced by immune and neural cells. In vivo studies with HSV-1 and WNV demonstrate the significance of specific cytokines (IFNγ and IL-1β, respectively) that modulate NSC responses during infection [100,105], and provide a crucial model for understanding the complexity of viral pathogenesis in the brain.

Currently, studies are underway to explore antiviral and anti-inflammatory drugs as tools to protect NSCs during viral infections. Atorvastatin, a competitive inhibitor of 3-hydroxy-3-methylglutaryl-coenzyme A (HMG-CoA) reductase, induces pro-survival signaling in NSCs through activation of the AKT and ERK-1/2 pathways [109]. In neonatal NSCs infected with JEV, atorvastatin protected the NSCs against apoptosis and preserved proliferation [108]. Anakinra, an antagonist of the IL-1 receptor, prevented learning deficits in a mouse model of WNV-induced cognitive dysfunction, where neurogenesis is impaired [100]. Together these studies highlight the importance of identifying specific pathways that are responsible for NSC dysfunction during viral infections in order to provide potential targets for therapeutic intervention.

## Figures and Tables

**Table 1:** Effects of viral infections on NSC proliferation, survival, and differentiation.

Virus	Model System/Cell Type	Effect on NSCs	Notes
**HSV-1**	Infection of adult mice or hippocampal NSC cultures from neonatal mice	Inhibits NSC proliferation [60]	Accumulation of amyloid β protein
	Human iPSC-derived NSC monolayer culture	Cell death [45]	
	Human iPSC-derived cerebral organoids	Moderate cell death compared to monolayer hNSC cultures [46]	Activation of pro-apoptotic genes
	Human iPSC-derived NSCs and cerebral organoids	Decreased expression of neuronal markers and increased astroglial markers [46]	
	Murine neonatal hippocampal NSCs	Inhibits neuronal and promotes astroglial differentiation [60]	
**HCMV**	Human fetal brain-derived NSCs	Inhibits NSC proliferation [34]	Disruption of Hes1 expression
	Human fetal NSCs	Induction of apoptosis of infected cells [30]	Unfolded protein response activation
	Human iPSC-derived NSCs	Induction of apoptosis [32]	
	Human fetal NSCs	Inhibits neuronal differentiation when infection occurs in first 24 h after induction of differentiation [30]	
	NSCs derived from human embryonic stem cells (ESCs)	Inhibits neuronal differentiation [85]	Via elevated PPARγ levels
**MCMV**	Murine embryonic cerebral stem cells	Reduction in NSC proliferation [31]	Inhibition of DNA replication
	Murine neonatal brain cell after intracerebral MCMV infection	Decreased neuronal numbers and decreased expression of immature neuronal markers [5]	
	Murine embryonic NSCs	Inhibits neuronal and astroglial differentiation but neuronal differentiation is more severely affected [31]	
**BDV**	Human fetal NSCs	Inhibits neuronal differentiation [2]	
	Human fetal NSCs expressing BDV phosphoprotein P or X protein	Inhibits neuronal differentiation [83]	Driven by BDV phosphoprotein P
**BVDV**	Bovine fetal NSCs	Inhibits astroglial differentiation [84]	
**EV71**	Murine brain-derived NSCs	Induction of apoptosis [38]	
**CVB3**	CVB3-infected neonatal mice	Induction of apoptosis and inhibition of proliferation of SVZ NSCs [39,40]	
**JEV**	JEV-infected neonatal mice	Loss of actively proliferating NSCs in the SVZ, impaired proliferation in vitro. [4]	
	Human fetal NSC culture or NSCs isolated from brain of JEV-infected patients	Induction of apoptosis [42]	Increased expression of pro-apoptotic proteins and factors associated with ER stress. Increased cleavage of caspases-3, 7, 8, and 9.
	JEV-infected neonatal mice	Induction of apoptosis in the SVZ [108]	Reversed by atorvastatin treatment
	Murine neonatal SVZ NSCs	Inhibits neuronal and glial differentiation [1]	Downregulation of neuronal genes and decreased STAT3, JAK1, and increased STAT1 expression
**LCMV**	LCMV-infected neonatal mice	Increased cell death in the SVZ during adulthood [37]	
	Adult mouse brain harvested after neonatal LCMV infection	Decreased neuroblasts in the SVZ and SGZ [37]	Associated with chemokine expression
	Murine adult hippocampal and SVZ cells after congenital LCMV infection	Decrease in adult hippocampal neurogenesis [96]	
**WNV**	Human fibroblast iPSC-derived NSCs	Induction of apoptosis [41]	
**USUV**	Human fibroblast iPSC-derived NSCs	Induction of mild apoptosis of NSCs compared to WNV [41]	
**HIV**	Human NSCs treated with HIV gp120	Reduced proliferation and induction of quiescence.	Reduced Erk phosphorylation
	Hippocampal tissue from HIV patients	Reduced NSC numbers [59]	
	Adult murine hippocampal NSCs or adult mice treated with HIV gp120	Inhibition of NSC proliferation [33]	Cell cycle arrest in the G1 phase via MAPK pathway activation
	Fetal hNSCs treated with HIV tat	Increase in quiescent NSCs [36]	Increased nuclear localization of TRIM32 due to increased miR-155
	Adult brain tissue from HIV seropositive patients		
**ZIKV** African strain (MR766)	Human iPSC-derived forebrain NSCs	Reduced cell proliferation [29]	DNA damage, activation of the DNA damage response (DDR), and cell cycle arrest in the S-phase
	Embryonic murine NSCs or human embryonic NSCs	Induction of apoptosis and autophagy [51]	Increases macroautophagy to promote viral replication and disrupts selective autophagy
	hNSC cell line	Induction of apoptosis [55]	Activation of DDR, increased phosphorylation of H2AX (cellular protein responsive to DDR), and increased PARP and cleaved caspase 3
African strain (IB H 30656)	Embryonic murine cortical NSCs	No effect on cell viability [52]	Cytopathic effect (alterations in NSC secretome)
	Embryonic murine NSCs	Downregulation of neuronal and oligodendroglial genes and upregulation of astroglial genes [52]	
American strain (PRVABC59)	Human iPSC-derived forebrain hNSCs infected with ZIKV	Reduction in proliferation [29]	DNA damage, activation of DDR, and cell cycle arrest in the S-phase
	Embryonic murine cortical NSCs	No effect on cell viability [52]	Mild DDR induction in comparison to MR766 strain and activation of p53
	hNSC cell line	Mild cytopathic effect (pyknotic nuclei) [55]	Poor innate immune activation in hNSCs
	Human fetal NSCs	Induction of apoptosis [57]	Persistent ZIKV infection
Brazilian strain (Paraiba)	Embryonic murine NSCs or human embryonic NSCs	Induction of apoptosis and autophagy in NSCs [51]	Increases macroautophagy to promote viral replication and disrupts selective autophagy
Brazilian strain (ZIKV-BR)	NSCs from human iPSCs from exfoliated deciduous teeth	Aberrant neuronal differentiation with downregulation of mature neuron markers and upregulation of immature neuron markers [86]	
Brazilian strain (BR_ZIKV_AB_ES)	Human iPSC-derived NSCs	Inhibits neuronal differentiation [88]	
French Polynesia starin (H/PF/2013)	Human iPSC-derived NSCs	No changes in cell viability [41]	Cleaved caspase-3
	Human iPSC-derived NSCs	Premature differentiation to neurons [87]	
American strain (FB-GWUH-2016)	Human iPSC-derived NSCs	Premature differentiation to neurons [87]	
ZIKV proteins (NS4A and NS4B)	Human fetal NSCs	Decreased neuronal and astrocytic numbers [91]	Inhibition of Akt-mTOR pathway

## Data Availability

Not applicable.
